# What If the Power of the Family Is Missing?

**DOI:** 10.1111/nicc.70238

**Published:** 2025-11-09

**Authors:** Anna‐Henrikje Seidlein

**Affiliations:** ^1^ Institute of Ethics and History of Medicine University Medicine Greifswald Greifswald Germany

**Keywords:** care ethics, discrimination, equity, moral distress, social determinants of health

## Abstract

This article explores ethical challenges surrounding patients without a family within the framework of family‐centred care (FCC) in intensive care units, exemplified by a case. Families function as powerful social determinants of health, and FCC is built on their existence and presence. Complications and poorer outcomes raise concerns about justice, equity and nurses' moral integrity for patients without strong family involvement. The latter risks disadvantages, while nurses might face moral distress when acting as their sole advocates. Care ethics is offered as a comprehensive tool for analysis and to identify specific measures to counteract inequality.

## 1 Clinical Scenario (Fictitious)

Mr. Johnson (82 years old) was admitted to the intensive care unit (ICU) following a massive stroke. He came to Germany as a migrant worker in 1964 and lives on his own (involuntarily alone) with no known family extant. After initial stabilisation, he developed pneumonia, delirium and became ventilator‐dependent. The ICU staff were unable to locate an advance directive or power of attorney. Nurse Anne, who had cared for him daily for 2 weeks, reported that he is stressed and agitated during (nursing) procedures. She felt that he was increasingly introverted and had given up on himself. However, she also observed that when she was able to take time to be with and talk to him, he calmed down, and she could sometimes succeed in reorienting him.

Mr. Schmidt, with a similar disease and comparable age and health status, was in the next room; however, he had daily visits from his best friend and grandchildren, who read to and sang for him and—after instruction and under supervision—also performed swallowing exercises with him. He experienced no complications and made good progress.

The senior physician prognosticated Mr. Johnson's outcome to be poor and announced the withdrawal of treatment as, in his view, the patient's quality of life was expected to be poor. Nurse Anne felt that the responsibility for advocating on behalf of Mr. Johnson, lacking a family, fell heavily on her. She was troubled by the uncertainty of his wishes and the sense that he was receiving less support than patients with family involvement. Anne realised she could not fully replace the role of the family. She was left with the conviction that his chances of recovery had been worse from the outset and that, despite her intentions, she was participating in the reproduction of social inequality.

## 2 Background: Family‐Centred Care

Family‐centred care (FCC) is a key element of patient‐centred care, which aims ‘to improve individual health outcomes’ [1]. While FCC is an important cornerstone of intensive care today [2], family members have been considered primarily as visitors at the ICU for decades, and their access has been strictly regulated [3]. FCC has been defined as ‘mutually beneficial partnerships between health care providers (HCPs), patients, and families in health care planning, delivery, and evaluation’ [4]. According to FCC, (objective) medical and nursing needs, moral values (i.e., honesty, principles such as justice) and norms (i.e., rules of conduct to ensure the realisation of values), as well as individual preferences of both patients and their families are at the centre of healthcare provision and guide its orientation [5]. In this context, the term family refers to related or unrelated individuals who have a meaningful, supportive relationship with the patient [6]. Within the framework of FCC, families are involved and see themselves as active ‘co‐carers’ [2]. The benefit of FCC, however, includes *all* parties involved: FCC cannot only improve the family members' outcomes, but also those of the *patients*' (e.g., reduced delirium rates [7]) and the *nurses*' (e.g., increased satisfaction) as well as other team members.

The crux is that FCC assumes that every patient has a supportive social family network—but this is not always the case. In reality, there are patients who do not (or no longer) have a family, either consciously by choice or involuntarily by circumstances. Examples are the ‘very old’ or ‘oldest old’ patients in the ICU who have lost their social network due to death, the ‘solo ager’ without children, or geographically distanced patients due to migration, to mention just a few. In addition, other constellations may complicate or hinder the integration of families from their own, the patient's and/or healthcare professionals' perspectives. These include, for example, families in which difficult power dynamics and imbalances [8], toxic relationships, dysfunctionality [9] or even violence (e.g., emotional/psychological, physical, sexual) play a role [10].

Hence, what happens to those patients *without* a (supportive) family at times of FCC? There is a lot at stake for them.

## 3 Main Ethical Concepts and Issues

### 3.1 Family as a Social Determinant of Health

‘The social determinants of health [SdoHs] are the non‐medical factors which influence health outcomes’ [11]. There is a lively debate within the context of ICU treatment and some evidence about the potential influence of, for example, race, ethnicity, gender and income as SDoHs regarding decision‐making, rehabilitation and health service delivery [12, 13, 14, 15, 16].

However, families are also crucial for recovery, not only during but also after the ICU stay. Nevertheless, little has been said about how the families as SDoHs impact the health trajectories. Moreover, evidence suggests that SDoHs (and their interaction) are currently not systematically captured in intensive care medicine [17].

Starting from the premise that families are a powerful and important SDoH [18, 19], several ethical issues arise concerning the case described which have important implications for the provision of intensive care.

### 3.2 Social Isolation: Stereotypes and Discrimination

Social isolation (the quantifiable lack of social contacts)—as described in Mr. Johnson's case—is a growing problem, especially among older [20] and immigrant adults [21]. It is considered an independent factor for increased mortality and disability [22]. Social isolation can lead to stigma [23] and discrimination. Conversely, discrimination and stigmatisation may also result in social isolation [24]. This reciprocal relationship creates a vicious circle that further increases health‐related inequality. What is particularly relevant in this context is that discrimination resulting from different social categories overlaps and reinforces each part (so‐called ‘intersectionality’ [25]). This intersectional perspective captures the complex multiple discrimination, which, in the present case, could manifest in stereotypes about older people (so‐called ‘ageism’) and socially isolated people (e.g., personal failure). These stereotypes can lead to (implicit or explicit) bias in decision‐making. Respecting the patient's right to self‐determination is central, but Mr. Johnson cannot express his wishes. If there are no reliable sources for evaluating his will and treatment preferences and members of the family as guardians and mediators as well as decision‐making proxies are missing, there is a higher risk that implicit assumptions (e.g., a life without a social environment is less worth living) will influence the medical indication setting and treatment choices that can lead to over‐ or undertreatment of the patient. The senior physician's assessment that Mr. Johnson's quality of life would be poor may reflect such an implicit bias rather than the result of a ‘best‐interest’ assessment and evaluation.

### 3.3 Health‐Related Inequality and Injustice

To ‘Leave No One Behind’ is a health‐related goal in the United Nations' 2030 Agenda for Sustainable Development [26]. Justice is one of nurses' central values, which are strongly grounded in human rights [27]. The ethos of nursing, as set out in the International Council of Nurses' Code of Ethics, calls for nurses to ‘advocate for equity’ [27]. Equity ‘sets a standard or bar to guide […] efforts toward a collective future where people […] are able to live with dignity and reach their full potential, without systemic disadvantage associated with social, economic, geographic or environmental conditions’ [28]. It has been recently discussed in intensive care [29], but not in relation to the family as an SDoH and its significance for FCC. The fact that health disparities in the ICU do indeed exist was also recently impressively documented [30].

In the case illustrated above, Mr. Johnson appears to achieve different outcomes compared to Mr. Schmidt, likely due, at least in part, to the absence of a family.^1^ Thus, he is receiving different care. This disparity challenges the ethical principle of justice. Consequently, the question arises whether patients without family and social support may be at an inherent disadvantage in FCC environments, even though their medical needs are comparable. It is, therefore, important to emphasise that equity does not mean the *same* treatment for everyone, but rather, in view of the different resources and capabilities of each individual, it may even mean more and/or different treatment. Yet, it is unclear what equal care looks like in this case and how it could be achieved. In any case, perpetuating existing health inequalities should be avoided.

### 3.4 Nurses' Moral Integrity and Moral Suffering

Patients with families have someone who stands up for their values; they have a stronger lobby for their wishes and treatment preferences than those without a family. Nurse Anne feels morally burdened by being Mr. Johnson's only advocate and perceives that his care is disadvantaged without family support. She feels remorse and guilt as signs of moral distress, which compromise her moral integrity [31]. According to the original, narrow understanding of moral distress, it occurs when a nurse knows what they believe is the right action but feels constrained from acting [32]. Following a broader understanding of moral distress with several subcategories, it can also occur when a nurse is ‘unable to choose between two or more moral requirements’ and feels caught in a dilemma due to competing obligations (so‐called ‘moral dilemma distress’ [33]). Even if the attempt to replace the family can be seen as going beyond what is necessary (a so‐called ‘supererogatory act’), the nurse's experience cannot be dismissed, as she experiences moral distress resulting from her inability to act in a manner consistent with her ethical values and professional standards. Of course, the care provided by a family cannot be compensated for, and a line must be drawn regarding the duties of nurses, as it cannot be expected that they will act in a supererogatory manner. Nevertheless, the feeling of having to substitute for care, make decisions without the feedback of a family and bear the burden of responsibility alone can result in additional stress that must be considered.

### 3.5 Comprehensive Reading Through Care Ethics

A care ethical perspective could offer an additional layer to a more comprehensive understanding of Mr. Johnson's case. Care ethics is a theoretical approach that focuses on the reflection of power (asymmetries) and responsibility and aims to reveal and overcome inequality [34]. It places relationships, context and vulnerability at the centre of moral reflection. It assumes that humans are always—more or less—mutually dependent and autonomy is relational rather than individualistic. Care ethics is deeply rooted in feminist tradition but has developed into diverse (normative) theories. However, the consideration of intersectionality is also part of the philosophy of many care ethical theories.

Through this lens, a comprehensive ethical reflection is possible: Mr. Johnson's autonomy is not only limited by his acute illness but also his vulnerability is increased by age, migration history and lack of family. His exceptional vulnerability creates a duty for nurses (and other healthcare professionals) to ensure that his voice is acknowledged in decisions. Care ethics calls for individuals to take responsibility and develop concrete solutions to respond to ethical issues and moral adversity. Regarding Mr. Johnson's case, it could result in shifting the focus from abstract principle‐based discussion (e.g., What does equity mean?) to ask ‘How can (nursing) care practices sustain dignity for those who lack relational support through a family?’ Concerning Nurse Anne's attentiveness, care ethics could then lead to the question of what might be useful measures to shoulder the (moral) burden more between nurses and protect and strengthen Anne's moral integrity when faced with such a challenge. This could raise the awareness of tools such as peer support and interprofessional ethical reflection.

## 4 Implications for Critical Care Nurses

Mr. Johnson's case reveals a structural limitation of FCC, namely, when patients are socially isolated. To be sure, it is not the primary task of intensive care medicine to compensate for social inequality, but good intensive care recognises when social factors jeopardise the success of treatment and responds to them in a structured, transparent and interprofessional manner. Applied to Mr. Johnson's case, this could mean, that, through a structured, interprofessional case discussion, the expertise and experience of the team members are gathered, pooled and used to generate further ideas to support individual recovery. Possible results could include for example the involvement of volunteers (see, e.g., [35]) to allow more time for personal dialogue and interaction. The involvement of clinical ethics consultants, nurse ethicists or other structures of clinical ethics or individuals with such a mandate (see, e.g., [36, 37]) could also be helpful in order to provide moral relief for nurses and other healthcare professionals on the one hand and to ensure adequate patient representation on the other. At a more general level, special attention could also be paid to enhance the team's knowledge and raise awareness of unconscious bias against patients who do not have a family and related challenges as well as to develop a guideline for the ward/institution, on what needs to be taken into account in order to best meet the needs of these patients under the given circumstances.

There is a need to look at FCC frameworks from a viewpoint that aims to ensure they are inclusive and adaptive to patients without families in order to avoid FCC high‐quality intensive care becoming family‐dependent care.

Communication and empathy, for example, play a central role, and a patient without relatives may need more time for attention and conversations with caregivers.

In order to ensure that intensive care is compassionate and appropriate for all patients, it is necessary to establish structures that also work for patients without a supportive social network.

## Ethics Statement

The author has nothing to report.

## Consent

The author has nothing to report.

## Conflicts of Interest

The author declares no conflicts of interest.

## Data Availability

Data sharing not applicable to this article as no datasets were generated or analysed during the current study.

## Endnotes

^1^
There may, of course, be other individual factors and social determinants of health besides family that influence recovery, for example, illness perception, personality and level of education.
